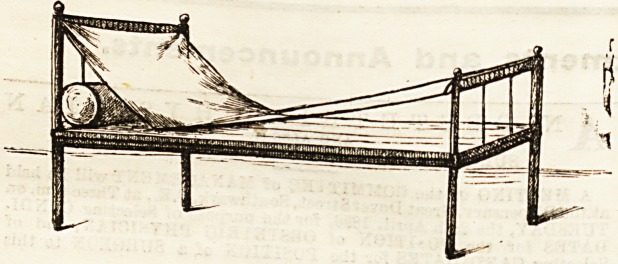# Practical Departments

**Published:** 1896-04-11

**Authors:** 


### PRACTICAL DEPARTMENTS.

### PILLOW SLING.

The Pillow Sling sketched below is the happy invention of a lady, Miss Weatherly, of Hill Side, Portishead, Somerset. The idea is simple enough, and owes its origin to Miss Weatherly's personal experience of the needs of an invalid. The sling consists of a piece of canvas to which are securely attached straps at the four corners, two short ones with buckles at the top, and two long ones provided with slip nooses at the bottom. With these straps the sling is fastened to the head and foot rail of the bed, and with the pillow resting on the sling a most comfortable support is attained for the patient. The two long straps are a help in enabling the invalid to raise himself to a sitting position. It would be difficult to arrive at a more really restful support than this, firm and at the same time yielding to any change of posture. Wherever it has been in use it has been found to give very great satisfaction and comfort, especially in the case of sufferers from asthma. One invalid derived so much comfort from its use that he has in consequence presented several "Slings" to the Liverpool Infirmary. They may be obtained from Messrs. G. Lipsett and Co., who are the sole makers, 21, Leece Street, Liverpool. Prices 7s. 6d. and 10s. 6d.

## Figures and Tables

**Figure f1:**